# Experimental observation of ferrielectricity in multiferroic DyMn_2_O_5_

**DOI:** 10.1038/srep03984

**Published:** 2014-02-05

**Authors:** Z. Y. Zhao, M. F. Liu, X. Li, L. Lin, Z. B. Yan, S. Dong, J. -M. Liu

**Affiliations:** 1 Laboratory of Solid State Microstructures, Nanjing University, Nanjing 210093, China; 2 Department of Physics, Southeast University, Nanjing 210189, China

## Abstract

One of the major breakthroughs associated with multiferroicity in recent years is the discovery of ferroelectricity generated by specific magnetic structures in some magnetic insulating oxides such as rare-earth manganites RMnO_3_ and RMn_2_O_5_. An unresolved issue is the small electric polarization. Relatively large electric polarization and strong magnetoelectric coupling have been found in those manganites of double magnetic ions: magnetic rare-earth R ion and Mn ion, due to the strong R-Mn (4*f*-3*d*) interactions. DyMn_2_O_5_ is a representative example. We unveil in this work the ferrielectric nature of DyMn_2_O_5_, in which the two ferroelectric sublattices with opposite electric polarizations constitute the ferrielectric state. One sublattice has its polarization generated by the symmetric exchange striction from the Mn-Mn interactions, while the polarization of the other sublattice is attributed to the symmetric exchange striction from the Dy-Mn interactions. We present detailed measurements on the electric polarization as a function of temperature, magnetic field, and measuring paths. The present experiments may be helpful for clarifying the puzzling issues on the multiferroicity in DyMn_2_O_5_ and other RMn_2_O_5_ multiferroics.

Multiferroics have been intensively investigated for ten years since the pioneer works on BiFeO_3_^1^ and TbMnO_3_ in 2003^2^. In particular, the discovery of magnetically induced ferroelectrics (the so-called type-II multiferroics) has comprehended our understanding of multiferroicity^2^,^3^,^4^. In these materials, electric polarization *P* is believed to be correlated with particular magnetic orderings below certain temperatures and thus the cross-coupling between ferroelectricity and magnetism is significant, allowing possible magnetic control of ferroelectricity or/and electric control of magnetism^5^,^6^,^7^,^8^,^9^,^10^,^11^. To dates, what keeps the research interest alive is the possibility of unveiling microscopic physics which is substantially different from our earlier knowledge and even general principles for guiding the design and synthesis of multiferroics of promising practical applications^5^,^12^,^13^,^14^,^15^. It is noted that most discovered type-II multiferroics so far either have low ferroelectric and magnetic transition temperatures or exhibit small electric polarization/weak magnetization.

While conventional ferroelectrics exhibit the electric polarization via the structural symmetry-breaking transitions from high symmetric paraelectric (PE) phase^16^, for those type-II multiferroics the primary order parameter is magnetic rather than structural. Two major magnetic mechanisms for the ferroelectricity generation have been proposed. One is the asymmetric exchange striction, in which the inverse Dzyaloshinskii-Moriya (DM) interaction associated with the non-collinear spin ordering drives the structural symmetry-breaking^6^,^17^,^18^. The other is the symmetric exchange striction, in which specific collinear spin ordering, such as the E-type antiferromagnetic (AFM) ordering^19^,^20^ and ↑↑↓↓ ordering^21^, drives the structural symmetry-breaking.

Interestingly, another class of multiferroics, in which the symmetric exchange striction is believed to play major roles, is rare-earth manganites RMn_2_O_5_ family^22^. The RMn_2_O_5_ family shows complicated lattice distortions and spin structures, and exhibits multifold competing interactions, large electric polarization, and remarkable magnetoelectric (ME) responses^5^,^16^,^23^. Nevertheless, partially due to the multifold competing interactions, the multiferroic transitions and underlying mechanisms in RMn_2_O_5_ are not yet well understood^8^,^9^. All members of this RMn_2_O_5_ family have similar structural ingredients^16^. The lattice structure projected on the *ab*-plane is shown in Fig. 1. The Mn ions are partitioned into Mn^3+^ and Mn^4+^, which are coordinated respectively in square pyramid Mn-O units and octahedral Mn-O units. On the *ab*-plane, the octahedra and pyramids are corner-sharing by either the pyramid bases or pyramid apex, and the adjacent pyramids are connected with their bases. Along the *c*-axis, the octahedral sharing edges constitute linear chains. Each Mn^3+^ ion is located in between two Mn^4+^ ions, and the R^3+^ ions are located on the alternative layers between two Mn^4+^ ions.

In RMn_2_O_5_, the Mn spin interactions are characterized by the three dominant components *J_3_*, *J_4_*, and *J_5_*, plus additional long-range components^16^,^24^. Their competitions lead to consecutive commensurate antiferromagnetic (C-AFM) and incommensurate AFM (IC-AFM) ordering sequence^25^,^26^. Also, the 3*d*-4*f* interactions can't be neglected if the R ion has big moment, and the strong R-Mn coupling allows even more fascinating spin structure evolution. The ferroelectricity and its magnetic origins are thus far from understanding. Here, we choose DyMn_2_O_5_ as a representative example for illustration^8^,^9^,^24^. The paramagnetic phase above temperature *T* ~ 43 K transits into an IC-AFM phase, followed by a C-AFM phase below *T_N1_* = ~40 K, and then by the coexistence of an IC-AFM phase and a C-AFM phase below *T_N2_* ~ 28 K. This coexistence is again replaced by two coexisting IC-AFM phases below *T_N3_* ~ 20 K. At *T* < *T_Dy_* ~ 8 K, the Dy^3+^ spins order independently. The structural and interaction origins for these magnetic transitions were discussed extensively, while no full consistency has been reached^8^,^9^,^25^.

With respect to the magnetic structures, our understanding of the ferroelectricity is even in the earlier stage. While it is believed that the C-AFM phase is ferroelectric and the IC-AFM phase is not, the measured results are not always consistent with this prediction^8^,^9^. Basically, the measured *P* most likely aligns along the *b*-axis. However, the measured data in experiment by Hur *et al* show that the *P* appears below *T_N1_* (*T_FE1_*) and changes its sign from negative value to positive one at a certain *T* lower than *T_N2_* ~ 27 K, giving a feature of ferrielectric (FI) state^8^. However, in experiment by Higashiyama *et al*^9^, the measured *P*(*T*) experiences several transitions which correspond one-to-one to the magnetic transitions, and the system becomes non-ferroelectric below *T_Dy_* ~ 8 K (the so-called X-phase) while the ferroelectric nature of this X-phase remains unclear. Besides, the magnetic origins for these transitions were discussed in details^16^. So far available data on the ME effect of RMn_2_O_5_ family are also materials-dependent. Remarkable ME response was observed in the low-*T* range for those materials with big 4*f* magnetic moments, where the 3*d*-4*f* (R-Mn) coupling is strong enough in determining the magnetic structure^8^,^9^,^12^,^26^,^27^.

The inconsistencies and insufficient data, as partially highlighted above, suggest a critical appealing for revisiting the electric polarization and its response to magnetic field in RMn_2_O_5_ (here DyMn_2_O_5_). On the basis that DyMn_2_O_5_ has strong Dy-Mn interactions in addition to the dominant Mn-Mn interactions^28^, one has reasons to expect a ferrielectric state with more than one polarization component. With no doubt, convincing evidences with this ferrielectric state become primarily critical for understanding the multiferroicity of DyMn_2_O_5_ and more generally the RMn_2_O_5_ family. In this work, it will be suggested that DyMn_2_O_5_ is a ferrielectric composed of two anti-parallel ferroelectric sublattices. The electric polarizations of the two sublattices have different microscopic origins, with one arising from the Mn-Mn symmetric exchange striction and the other from the Dy-Mn symmetric exchange striction. It is noted that the ME effect can be reasonably explained by this ferrielectric model. We employ a modified pyroelectric current (mPyro) method to track the evolution of the electric polarization upon various paths, while a detailed discussion on the methodology for measuring the polarization and a description of this mPyro method can be found in the Supplementary document.

## Results

### Multiferroic phase transitions

**Figure 2:**
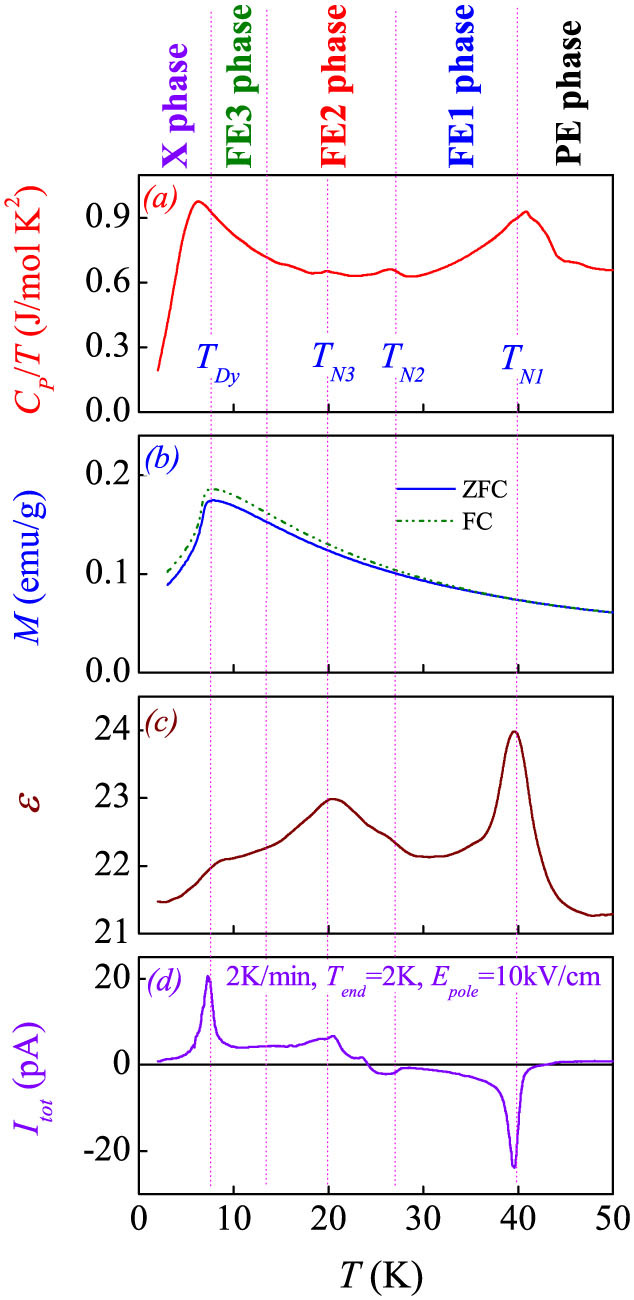
(a) Specific heat normalized by temperature (*C_P_*/*T*), (b) magnetizations (*M*) under the ZFC and FC conditions, (c) dielectric constant (*ε*), and (d) released current (*I_tot_* = *I_pyro_*) by the mPyro method at a warming rate of 2 K/min with a poling electric field *E_pole_* = 10 kV/cm, as a function of *T*, respectively. The dielectric constant was measured at frequency of 100 kHz with a bias of 50 mV, and no remarkable frequency dispersion was observed. The phase regions proposed in literature are labeled on the top.

We first look at the phase transition sequence in terms of specific heat *C_P_*, magnetization *M*, and dielectric constant *ε* as a function of *T*, as shown in Fig. 2. For reference, the released current *I_tot_*(*T*) (i.e. *I_pyro_*(*T*)) using the mPyro method at a warming rate of 2 K/min is shown in Fig. 2(d), while a demonstration of the mPyro method in precisely measuring the pyroelectric current released from polarized charges is given in the Supplementary document. The *C_P_*(*T*) curve shows clear anomalies roughly at *T_N1_* ~ 40 K, *T_N2_* ~ 27 K, and *T_Dy_* ~ 8 K, while the peak at *T_N3_* ~ 20 K, if any, is weak. These anomalies reflect the sequent magnetic transitions from the paramagnetic phase to the C-AFM phase, to the coexisting IC-AFM phase plus C-AFM phase, then to the two IC-AFM coexisting phases, and eventually to the independent Dy^3+^ spin order plus IC-AFM phase, consistent with earlier reports^26^. However, no features corresponding to these transitions, except the independent Dy^3+^ spin ordering at *T_Dy_*, were observed in the measured *M-T* data, mainly due to the fact that the Dy^3+^ moment is much bigger than the Mn^3+^/Mn^4+^ moments. The anomalies in the *ε*-*T* curve at these phase transitions reflect the magneto-dielectric response, as revealed earlier^8^. Interestingly, a series of anomalies in the *I_pyro_*-*T* curve at these transition points are available, as shown in Fig. 2(d), consistent with earlier report^8^ too, evidencing the strong ME effect. In addition, these magnetic transitions may be path-dependent, and the features in the cooling run are different from those in the warming run. Our data on the electric polarization below also illustrate this dependence.

### Nonzero polarization of the X-phase

**Figure 3:**
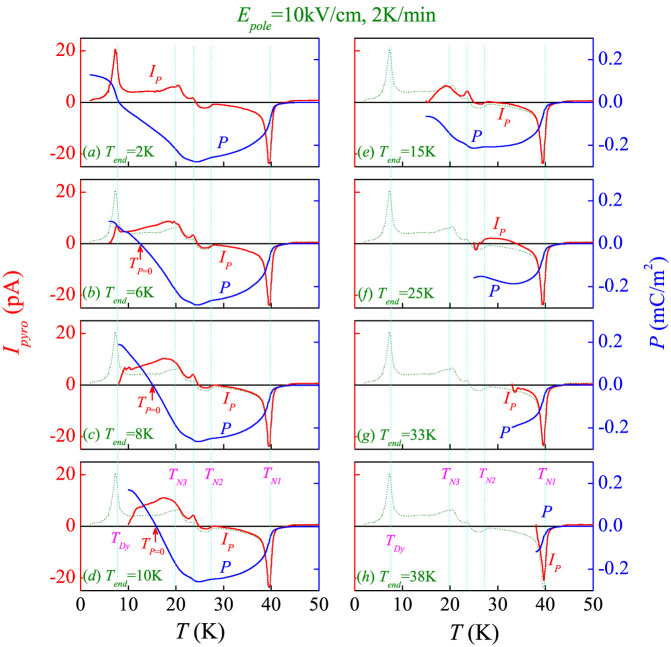
Measured pyroelectric current *I_pyro_* = *I_P_* and evaluated electric polarization *P* as a function of *T* at *T_end_* = 2 K (a), 6 K (b), 8 K (c), 10 K (d), 15 K (e), 25 K (f), 33 K (g), and 38 K (h), respectively. The warming rate is 2 K/min. For reference, the *I_P_*-*T* data at *T_end_* = 2 K are inserted.

The measured *I_pyro_*-*T* curves and as-evaluated *P-T* curves under *E_pole_* = 10 kV/cm are plotted in Fig. 3, given different starting temperatures (*T_end_*), where the *T_end_* is the temperature to which the sample is cooled down from high-*T* paramagnetic state under *E_pole_* = 10 kV/cm. It is seen that the *I_pyro_*-*T* curves exhibit clear anomalies at the magnetic transition points (*T_N1_*, *T_N2_*, *T_N3_*, and *T_Dy_*), and the *P-T* curve at *T_end_* = 2 K is similar in shape to that reported in Ref. 8.

We suggest that the X-phase is ferrielectric (i.e. ferroelectric in general sense). First, the measured data in Fig. 3(a) and (b) indicate that the X-phase has nonzero electric polarization. The pyroelectric current in both the X-phase region and the other three ferroelectric regions is much bigger than 0.3 pA, the background level in the present experiment. It is also observed that the pyroelectric current depends on the poling field, the bigger the current the higher the field. The mPyro method used in the present experiment is different from the Pole method used in Ref. 9 for the electric polarization measurement (see the Supplementary document). In the Pole method, the current flowing across the sample under a relatively low electric field is measured during the sample cooling. In this case, by assuming that the leakage current at low *T* is much lower than the polarization current, one may evaluate the electric polarization from the polarization current data directly. As seen in Ref. 9, the measured polarization in the X-phase region was indeed negligible, suggesting that the X-phase is non-ferroelectric. Clearly, if the X-phase is antiferroelectric, or ferrielectric with two comparable antiparallel polarization components, the polarization current can be small and even comparable with the leakage current. In this sense, the Pole method may not be applicable for identifying the ferrielectric or antiferroelectric state.

Second, each measured *P*(*T*) curve shown in Fig. 3(a) ~ (d) indicates a negative-positive sign change with decreasing *T*, suggesting immediately that DyMn_2_O_5_ is a ferrielectric (FI) rather than a normal ferroelectric^8^. The sign change would be the consequence of competition between the two ferroelectric sublattices whose polarization components should exhibit different *T*-dependences. In this case, careful measurement using the mPyro method can provide critical data on details of the ferrielectric state and the different *T*-dependences.

### Path-dependent polarization

**Figure 4:**
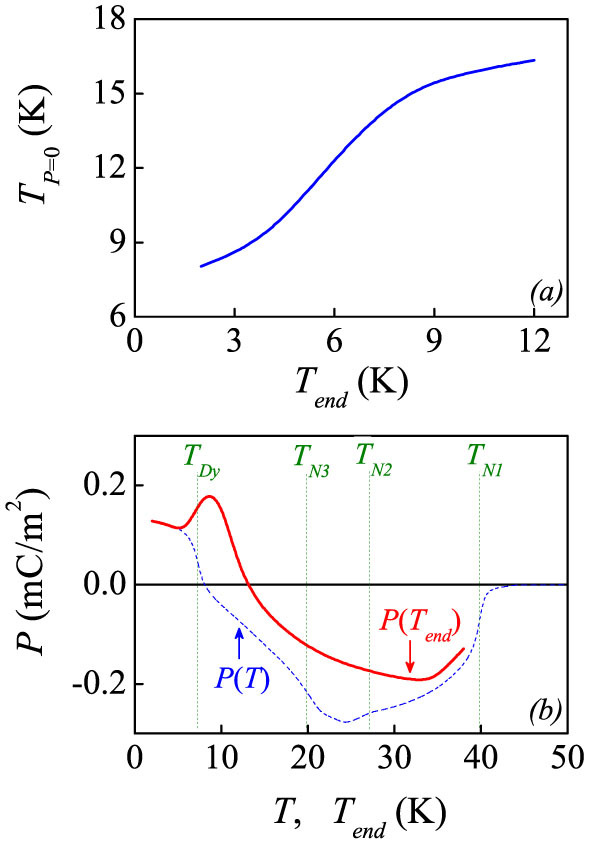
(a) Evaluated crossing temperature *T_P_*_ = 0_ at which the measured *P*(*T*) changes its sign, as a function of *T_end_*. (b) Evaluated *P*(*T*) curve and *P*(*T_end_*) curve. The warming rate for the pyroelectric current probing is 2 K/min.

Given a fixed *E_pole_*, the *I_pyro_*(*T*) and thus the *P*(*T*) show remarkable *T_end_*-dependent behaviors, i.e. path-dependent. To see clearly this path-dependence, the *I_pyro_*-*T* curve with *T_end_* = 2 K is shown by a thin dashed line in each plot of Fig. 3. The temperature *T_P_*_ = 0_, at which the *P*(*T*) changes its sign, is plotted as a function of *T_end_* in Fig. 4(a). The *T_P_*_ = 0_ shifts ~9 K when *T_end_* increases for ~10 K, implying that the ferrielectric state is not robust against thermal fluctuations (*T*) or external field (*E_pole_*). Supposing that the ferrielectric state is composed of two sublattices, one expects that at least one of them is strongly *T*-dependent or *E_pole_*-dependent. What surprising us is the *I_pyro_*-*T* curves as *T_end_* > 12 K, some of which are plotted in the right column of Fig. 3. In spite of positive *E_pole_*, the measured *P* data below *T_N1_* are negative. The negative *I_pyro_* peak at *T_N1_* remains nearly unchanged even with *T_end_* = 38 K, very close to *T_N1_* = 40 K. Such a negative *P* can't be possible in a normal ferroelectric, unless the *P* has two components which are anti-parallel to each other.

The *T_end_*-dependences of the *I_pyro_*(*T*) and *P*(*T*) curves are strange at the first glance. It is reflected that the electric polarization has the magnetic origin, since no structural phase transitions occur below *T_N1_*. We present in Fig. 4(b) the measured *P* value at *T_end_*, i.e. *P*(*T_end_*), where the *P*(*T*) curve with *T_end_* = 2 K is inserted for comparison. For a normal ferroelectric, the *P*(*T_end_*) should overlap with the *P*(*T*) by setting *T_end_* = *T*. Here, the overlapping only occurs below *T_Dy_*, noting that the *P*(*T_end_*) is always larger than the *P*(*T*). The difference between them maximizes at *T_end_* = *T* ~ 10 K and ~24 K and becomes negligible as *T_end_* → *T_N1_*, suggesting that the magnetic transitions below *T_N1_* have the path-dependent characteristic, while the first-order or second-order nature of these transitions deserves for additional clarification. In fact, combining the *P*(*T_end_*) and *P*(*T*) data generates a double-loop like hysteresis, as shown in Fig. 4(b).

## Discussion

For DyMn_2_O_5_, the symmetric exchange striction effect arising from the specific Mn/Dy spin alignment is the main mechanism for the electric polarization^16^. For a simplification consideration, we don't take into account the contributions from the noncollinear spin orders. However, the effect of the independent Dy spin ordering at *T_Dy_* imposes significant effect on the electric polarization, due to the strong Dy-Mn interactions, and thus will be considered.

**Figure 5:**
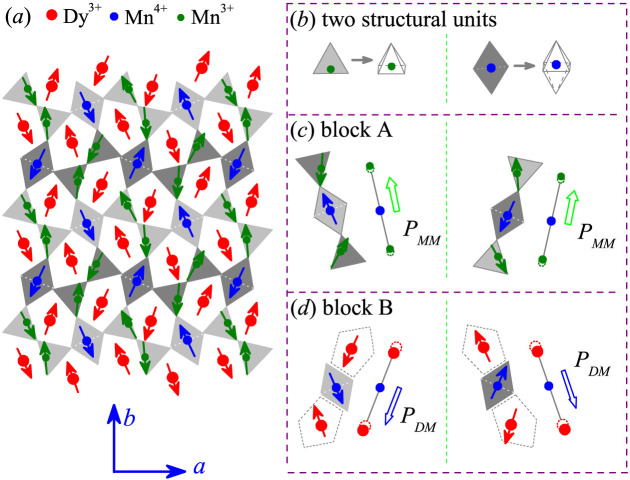
Proposed spin structure at a temperature lower than *T_N2_* and higher than *T_Dy_*, referring to neutron scattering data available in literature. (a) The spin structure projected on the *ab*-plane with the square pyramidal Mn^3+^-O^2−^ unit and octahedral Mn^4+^-O^2−^ unit shown in (b). The structural block A, composed of one Mn^4+^-O^2−^ octahedra connected by two Mn^3+^-O^2−^ pyramids roughly along the *b*-axis, is shown in (c). The structural block B, composed of one Mn^4+^-O^2−^ octahedra connected by two Dy^3+^ roughly along the *b*-axis, is shown in (d). The proposed polarizations *P_MM_* and *P_DM_* generated by the two types of blocks due to the symmetric exchange strictions, are labeled in (c) and (d), respectively.

Referring to relevant literature on DyMn_2_O_5_^24^, we present in Fig. 5(a) the spin structure projected on the *ab*-plane over the *T*-range between *T_N1_* and *T_Dy_*. The square pyramidal and octahedral structural units surrounding the Dy^3+^, Mn^3+^, and Mn^4+^ spins are drawn for a better view in Fig. 5(b). The light gray and gray structural units shift 1/4 lattice unit from each other along the *c*-axis^16^. Along the *b*-axis, one finds two types of three-spin blocks each centered on a Mn^4+^ spin, as shown in Fig. 5(c) and (d), respectively. One is block A, consisting of one Mn^4+^-O octahedron connected with two pyramid units each with one Mn^3+^ spin inside (Fig. 5(c)). The other is block B, consisting of one Mn^4+^-O octahedron connected with two Dy^3+^ spins located in the space surrounded by the MnO_6_ and MnO_5_ units (Fig. 5(d)). Because of the symmetric exchange striction, the two Mn^3+^ ions in the block A shift roughly up and the two Dy^3+^ ions in the block B shift down with respect to the Mn^4+^ ions. Therefore, one electric polarization component (*P_MM_*) in the block A and one polarization component (*P_DM_*) in the block B are generated. They are roughly anti-parallel to each other but align along the *b*-axis. The whole lattice as the consequence of the alternating stacking of the two types of blocks is therefore a ferrielectric lattice composed of two FE sublattices.

Different from the *P_MM_*, the *P_DM_* originates from the Dy-Mn interactions and thus depends on the Dy^3+^ spin order. Above *T_Dy_*, the Dy^3+^ spins may order in coherence with the Mn spin ordering around *T_N1_* or *T_N2_*, due to the strong Dy-Mn interactions. At *T* < *T_Dy_*, this induced Dy^3+^ spin ordering is partially and gradually replaced by the independent Dy^3+^ spin ordering, although the details of the Dy^3+^ spin ordering sequence has not been well understood. To this stage, one has reason to argue that the *P_DM_* will show much more significant *T*-dependence than the *P_MM_*. The reason can be discussed considering the weak ordering of the Dy^3+^ spins themselves. This ordering is sensitive to the 3*d*-4*f* interactions and external field. We discuss this issue below from various aspects.

First, the 4*f* interactions in some transition metal oxides are quite localized and the R^3+^ spins alone can't order unless the temperature is very low (the ordering point *T_R_* is less than ~2 K), as identified in oxides without 3*d* moments but only the 4*f* moments, as seen in R_2_Ti_2_O_7_ etc with R = Gd, Tb, Ho, and Er etc^29^, where the Ti^4+^ has no magnetic moment and thus no 4*f*-3*d* interaction is available. In some other oxides, the R^3+^ spins can't order even at extremely low temperature, leading to spin liquid or spin ice states due to the crystal fields and quantum fluctuations^30^,^31^. In these oxides, a magnetic field of ~1.0Tesla is sufficient to break the original spin orders and enforce the parallel spin alignment.

Second, for DyMn_2_O_5_ and other RMn_2_O_5_/RMnO_3_ with R = Gd, Ho, Er etc, the situation can be different since the R^3+^ spins coexist with the Mn spins. The independent R^3+^ spin ordering can occur at a *T_R_* as high as 6 ~ 10 K (here *T_Dy_* ~ 8 K). Furthermore, if the Mn spin ordering occurs well above *T_R_*, an additional R^3+^ spin ordering at a temperature higher than *T_R_* will be induced by the Mn spin orders due to the 4*f*-3*d* interactions. These observations suggest that the 4*f*-3*d* interactions can enhance the *T_R_* value if the sign of interaction is consistent with that of the 4*f*-4*f* exchange interaction^16^,^32^,^33^. A typical case is seen in DyMnO_3_, where the Mn^3+^ spins order antiferromagnetically at ~38 K and are locked in the noncollinear spiral order at ~20 K. Slightly below this locking point, the Dy^3+^ spins order in a coherent manner with the Mn^3+^ spin order. This induced Dy^3+^ spins order sustains until *T_Dy_* ~ 7 K at which the independent Dy^3+^ spin ordering enters^34^. In addition, a magnetic field of 1.0 ~ 2.0Tesla is sufficient to break the coherent and independent Dy^3+^ spin orders, while much higher field is needed to melt the Mn spin orders.

**Figure 6:**
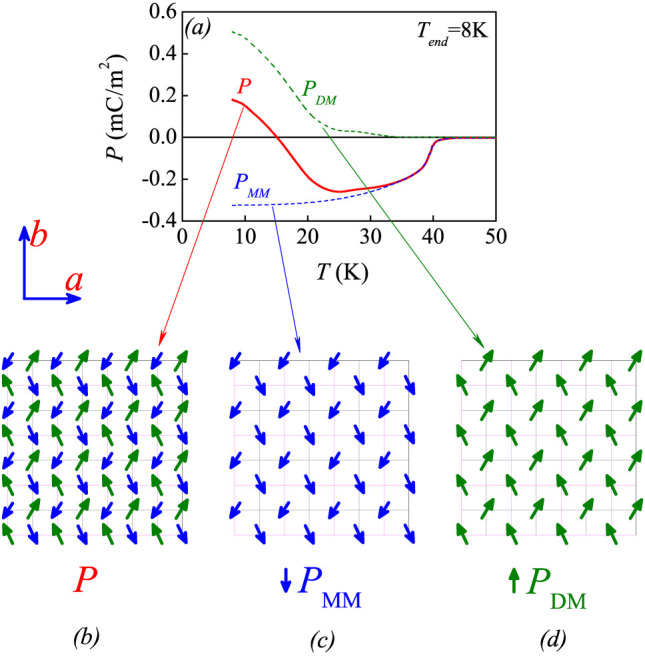
(a) Evaluated electric polarizations *P_MM_* and *P_DM_* from the two ferroelectric sublattices of the proposed ferrielectric model, as a function of *T*, where *P* = *P_DM_* + *P_MM_* and *T_end_* = 8 K. The proposed ferrielectric lattice and the associated two sublattices, all projected on the *ab*-plane, are schematically drawn in (b), (c), and (d), respectively.

The above discussion suggests that the Dy^3+^ and other rare-earth moments have relatively weak 4*f*-4*f* exchange-coupling with respect to the 3*d* moments such as Mn spins here. This discussion thus serves as the model basis on which the *P_DM_* and *P_MM_* as a function of *T* are evaluated, respectively. For simplification, the effect of independent Dy^3+^ spin ordering below *T_Dy_* on the Mn spin order is assumed to be weak if any. It can be reasonably assumed that the *P_MM_* initiating at *T_N1_* increases rapidly in magnitude with decreasing *T* and becomes saturated in the low *T* range, because the Mn spin order is already well developed below *T_N1_*. Consequently, the *P_DM_* as a function of *T* can be extracted. Take the data with *T_end_* = 8 K ~ *T_Dy_* as an example. The measured *P*(*T*) data are plotted in Fig. 6(a). The *P_MM_*(*T*) curve is extracted based on the above assumption, and then *P_DM_*(*T*) = *P*(*T*) − *P_MM_*(*T*) is evaluated. For a clear illustration, the two ferroelectric sublattices on the *ab*-plane are schematically drawn in Fig. 6(c) and (d), and a combination of them constitutes the ferrielectric lattice in Fig. 6(b). As expected, the *P_DM_* increases gradually with decreasing *T* until *T* ~ 20 K, below which a much more significant *T*-dependence than that for the *P_MM_* is then exhibited.

As *T_end_* = 2 K ≪ *T_Dy_*, the effect of the independent Dy^3+^ spin ordering on the *P_DM_* takes effect. At *T* = *T_end_*, some Dy^3+^ spins are on the track of the independent ordering, leading to disappearance of *P_DM_* at some lattice sites. The *P_DM_* sublattice is thus partially melted away, giving rise to a smaller *P_DM_*. This is the reason for the low *T*_P = 0_ and small |*P*| below *T*_P = 0_, with respect to the case of *T_end_* = 8 K. Here it should be mentioned that the difference in the *P_DM_*(*T*) curve between the case of *T_end_* = 8 K and that of *T_end_* = 2 K reflects the difference in the magnetic structures between the two cases. The origin lies in the fact that the magnetic transition at *T_Dy_* is path-dependent.

Given the ferrielectric model shown in Fig. 5 and the different *P_DM_*(*T*) and *P_MM_*(*T*) behaviors, a puzzling issue appears: why does the measured *P_MM_* (or *P*) remain negative even though *T_end_* is higher than *T_N2_*? For *T_end_* > *T_N2_*, the *P_DM_* should be much smaller than the *P_MM_* and thus a poling by a positive *E_pole_* would generate a positive *P_MM_*. In this case, the measured *I_pyro_* and *P* should be positive, contradicting with the measured data. At this stage, we have no convincing explanation of this anomalous phenomenon. One possible reason is that the *P_DM_*(*T*) is sensitive to the *E_pole_*. Considering the fact that the Dy^3+^ spins have weak exchange coupling, as addressed above, one expects that the electric field driven alignment of the Dy^3+^ spins coherently with the Mn spins would be energetically easy. Therefore, the *P_DM_* can be remarkably enhanced by the *E_pole_*. If it is the case, the electric poling during the cooling sequence can enhance the *P_DM_* remarkably while the *P_MM_* is roughly unchanged, so that the *P_DM_* around *T_end_* is larger than the *P_MM_* in magnitude. This results in the alignment of the *P_MM_* opposite to both the *E_pole_* and *P_DM_*. After the removal of the *E_pole_* at *T_end_*, the *P_DM_* shrinks back to a value smaller than the *P_MM_*. Consequently, the pyroelectric current remains negative. Another possible explanation for this strange phenomenon is the ferroelastic effect in DyMn_2_O_5_^35^, which makes the *P_MM_* domains be clamped along a direction opposite to the *E_pole_* during the poling process. However, this assumption remains to be confirmed.

**Figure 7:**
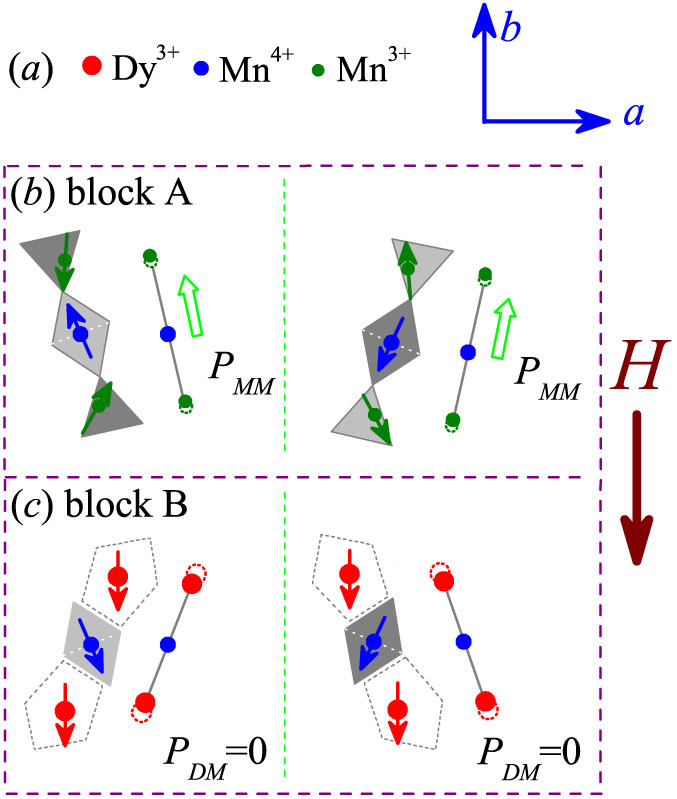
Schematic drawing of the spin alignments in the block A and block B, respectively, under a downward magnetic field *H* at *T* < *T_Dy_*. The Dy^3+^ spins can be easily re-aligned by *H* while the Mn spins can't, implying that the *P_DM_* = 0 at *T* < *T_Dy_*.

**Figure 8:**
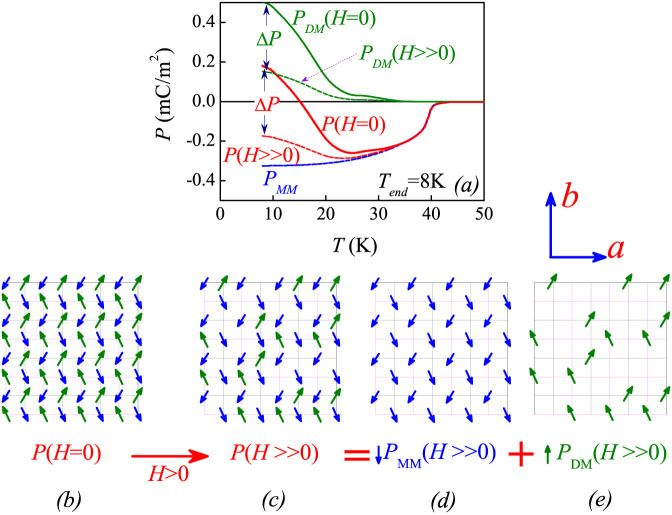
Measured ME responses and proposed model. (a) The measured *P*(*T*) curves and proposed *P_MM_*(*T*) and *P_DM_*(*T*) curves under *H* = 0 and *H* ≫ 0 (e.g. ~2 T). It is suggested that the *P_MM_* is robust against *H* while the *P_DM_* can be seriously suppressed by *H*, due to the field induced Dy^3+^ spin realignment as proposed in Fig. 7. The ferrielectric lattice at *H* = 0 is shown in (b), which transfers into the lattice in (c) at *H* ≫ 0. This lattice in (c) is composed of the *P_MM_* sublattice shown in (d) plus the *P_DM_* sublattice shown in (e). *P* = *P_DM_* + *P_MM_*.

Obviously, referring to the ferrielectric model, one immediately predicts that the ME parameter Δ*P*(*H*) = [*P*(*H*) − *P*(*H* = 0)] is negative and also remarkably *T*-dependent, since a magnetic field as big as 1.0–2.0Tesla is sufficient to align the R^3+^ spins even at an extremely low *T*^9^. For DyMn_2_O_5_ here, this effect suggests that a magnetic field of 1.0–2.0 Tesla re-aligns the Dy^3+^ spins along the field direction, while the Mn spins remain robust. Therefore, the ↑↑↓ or ↓↓↑ pattern in the block B is broken, as shown in Fig. 7, leading to the *P_DM_* ~ 0. The spin structure in the block A remains roughly unchanged and thus does the *P_MM_*. The experimental data conform this prediction, as presented in Fig. 8. In Fig. 8(a) are plotted the *P-T* data at *T_end_* = 2 K, where the *P_DM_*(*T*) and *P_MM_*(*T*) under *H* = 0 are presented too. The Δ*P*(*H* > 2 T) should not be much less than the *P_DM_* in magnitude although their signs are opposite. Our data also support this prediction. The ferrielectric state as a basis for this ME effect is then confirmed. Here, it should be mentioned that the model shown in Fig. 7 assumes that magnetic field *H* is parallel to the *b*-axis. Nevertheless, our samples are polycrystalline and the grains/magnetic domains are randomly oriented. Taking into account of the polycrystalline nature, one still can expect that the ↑↑↓ and ↓↓↑ patterns of most block B units in the sample will be broken. This model explanation is thus qualitatively reasonable.

To this stage, we have presented a qualitative explanation of the major features associated with the electric polarization and ME effect in DyMn_2_O_5_, based on the proposed ferrielectric model. Nevertheless, several issues remain yet unclear or unsolved: (1) No detailed discussion on the possible ferroelectric phase transitions at the magnetic transition points *T_N2_*, *T_N3_*, and even *T_Dy_*, respectively, has been given. (2) The path-dependence of the electric polarization is attributed to the magnetic transitions which are path-dependent. The first-order ot second-order nature of these magnetic transitions remains to be clarified. (3) An uncertain point regarding the present ferrielectric model is the response of the *P_DM_* to electric field which is assumed to be remarkable in order to account for the experimental observations. Searching for convincing evidence on this assumption is challenging although the assumption itself is physically reasonable. A careful characterization of the Dy^3+^ spin structures at various *T* is critical for dealing with these issues. It was reported that the element specific X-ray resonant magnetic scattering (XRMS) is a powerful tool although neutron scattering may face the problem of large absorption by Dy nuclei^36^.

In summary, extensive multiferroic measurements on DyMn_2_O_5_ have been carried out, and the complicated electric polarization behaviors have been characterized. It is revealed that the electric polarization in DyMn_2_O_5_ does consist of two antiparallel components, demonstrating the ferrielectric state at low temperature. The two electric polarization components are believed to originate from the symmetric exchange striction. One is generated from the Mn^3+^-Mn^4+^-Mn^3+^ blocks with the ↓↑↑ and ↑↓↓ spin alignments, which is robust against temperature and magnetic field. The other is generated from the Dy^3+^-Mn^3+^-Dy^3+^ blocks with the ↓↓↑ and ↑↑↓ spin alignments, which is sensitive to temperature and magnetic field. The present work represents a substantial step towards a full-scale understanding of the electric polarization in DyMn_2_O_5_ and probably other RMn_2_O_5_ family members.

## Methods

Polycrystalline DyMn_2_O_5_ samples were used for the present experiments. The samples were prepared by standard solid state sintering. Stoichiometric amount of Dy_2_O_3_(99.99%) and Mn_2_O_3_(99%) was thoroughly mixed, compressed into pellets, and sintered at 1200°C for 24 h in an oxygen atmosphere with several cycles of intermediate grindings. For every sintering cycle, the samples were cooled down to room temperature at 100°C per hour. The as-prepared samples were cut into various shapes for subsequent microstructural and property characterizations. The sample crystallinity was checked using X-ray diffraction (XRD) with Cu Kα radiation at room temperature.

Measurements on the specific heat (*C_P_*), magnetization (*M*) and *dc* magnetic susceptibility (*χ*), dielectric susceptibility (*ε*) and electric polarization (*P*) of the samples were carried out. All the data presented in this work were obtained from the polycrystalline samples. The *M* and *χ* were measured using the Quantum Design Superconducting Quantum Interference Device (SQUID) in the zero-field cooled (ZFC) mode and field-cooling (FC) mode, respectively. The cooling field and measuring field are both 1000 Oe. The *C_p_* was measured using the Quantum Design Physical Properties Measurement System (PPMS) in the standard procedure.

The polarization *P* was measured using the modified pyroelectric current (mPyro) method with different starting temperature *T_end_* = 2 K − 38 K, respectively. Each sample was polished into a thin disk of 0.2 mm in thickness and 10 mm in in-plane dimension, and then sandwich-coated with Au layers as top and bottom electrodes. The measurement was performed using the Keithley 6514 A and 6517 electrometers connected to the PPMS. In details, each sample was submitted to the PPMS and cooled down to ~100 K. Then a poling field *E_pole_* ~ 10 kV/cm was applied to the sample until the sample was further cooled down to *T_end_*, at which the sample was then short-circuited for sufficient time (>30 min) in order to release any charges accumulated on the sample surfaces or inside the sample. The recorded background current noise amplitude was ~0.3 pA. Then the sample was heated slowly at a warming rate up to a given temperature *T_0_* = 60 K > *T_N_*, during which the released current *I_tot_* was collected. Similar measurements were performed with different warming rates from 1 K/min to 6 K/min and the collected *I_tot_* data are compared to insure no contribution other than pyroelectric current *I_pyro_*. Finally, polarization *P*(*T*) was obtained by integrating the collected *I_pyro_*(*T*) data from *T_0_* down to *T_end_*. The validity of this procedure was confirmed repeatedly in earlier works^15^ and the data presented in the Supplementary document.

In addition, the *ε* data at various frequencies as a function of *T* were collected using the HP4294A impedance analyzer with an *ac-*bias field of ~50 mV. Besides the *ε*-*T* data and *P-T* and data, we also measured the response of *P* to magnetic field *H* in two modes. One is the isothermal mode with which the variation in *P* in response to the scanning of *H* was detected and the other is the iso-field mode with which the *P-T* data under a fixed *H* were collected. By such measurements, one can evaluate the ME coupling of the samples. We define Δ*P*(*H*) = *P*(*H*) − *P*(*H* = 0) as the ME parameter.

We also employed the PUND method to obtain the *P-E* loops at various temperatures, using the identical procedure as reported in literature e.g. Ref. 37. Our data are quite similar to reported ones from other groups (see the Supplementary document).

## Author Contributions

J.M.L. and Z.Y.Z. conceived and designed the experiments. Z.Y.Z., M.F.L., X.L., L.L., Z.B.Y. carried out the experiments. Z.Y.Z., S.D. and J.M.L. discussed the model. J.M.L. and Z.Y.Z. wrote the paper. All the authors discussed the results and commented on the manuscript.

## Supplementary Material

Supplementary Information Supplementary file

## Figures and Tables

**Figure 1:**
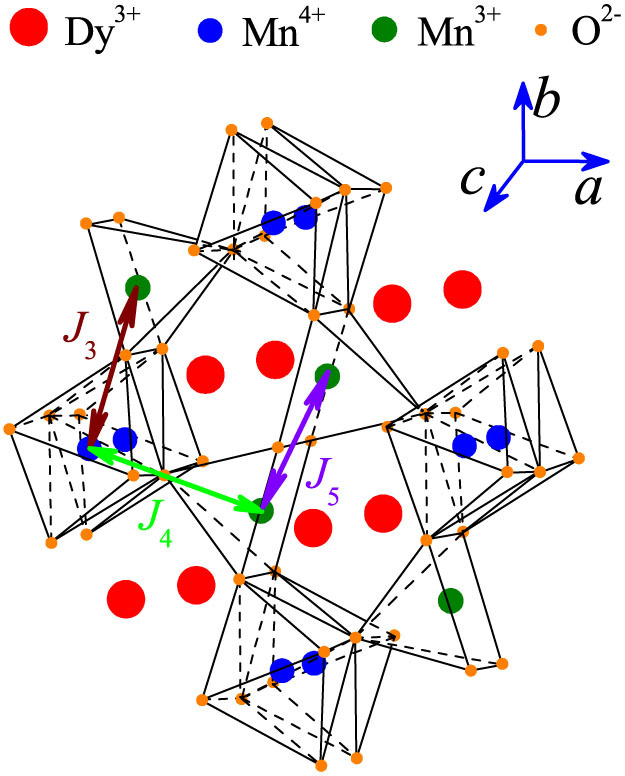
A schematic drawing of the lattice structure of DyMn_2_O_5_ with the three major Mn-Mn spin interactions *J_3_*, *J_4_*, and *J_5_*. The ions and coordinates are drawn for guide of eyes.
