# Supplementary material for: Experimental observation of ferrielectricity in multiferroic DyMn2O5
Source: Sci Rep. 2014 Feb 5;4:3984. doi: 10.1038/srep03984 (PMC3913922; doi:10.1038/srep03984)
Supplement: Supplementary Information — Supplementary file [file srep03984-s1.doc]

*Supplementary document for manuscript (Ref. SREP-13-04782)*

**Experimental observation of ferrielectricity in multiferroic DyMn_2_O_5_**

Z. Y. Zhao^1^, M. F. Liu^1^, X. Li^1^, L. Lin^1^, Z. B. Yan^1^, S. Dong^2^, and J. –M. Liu^1^

^1^*Laboratory of Solid State Microstructures, Nanjing University, Nanjing 210093, China*

^2^*Department of Physics, Southeast University, Nanjing 210189, China*

**I. Issues on methodology for measuring electric polarization**

*A. Methods for measuring polarization P*

So far available data on electric polarization *P* as a function of *T* and magnetic field *H* for type-II multiferroic materials were obtained by means of three different methods [1, 2].

We start from a discussion on the two methods employed for probing the electric polarization, and then present our data obtained by a modified pyroelectric current method, to be additionally complimented by the data obtained using the positive-up-negative-down (PUND) method.

The first method is conventional pyroelectric current (Pyro) method, which has been extensively used in measuring *P*(*T*, *H*) for multiferroics with extremely small polarization (as small as ~1.0μC/m^2^) and low transition temperature [1, 3]. The Pyro method is much more sensitive than the Saywer-Tower method or virtual-ground method conventionally used for the *P-E* hysteresis of normal ferroelectrics. A schematic illustration of the Pyro method is given in SFig.1(a). The sample covered with the top and bottom electrodes (e.g. Au), constituting a capacitor, is submitted to electric poling under a field *E_pole_* during the cooling run until *T*=*T_end_*, and then short-circuited for sufficient time at *T_end_*. The *T_end_* should be as low as possible and for typical case *T_end_*=2K. The released current *I_tot_* from the sample with electric bias *E_m_*=0 is probed during the subsequent warming run from the *T_end_* up to an assigned temperature *T_0_*. Certainly, the *T_0_* should be much higher than the highest ferroelectric transition point. If the released current *I_tot_* only contains the pyroelectric current *I_pyro_* without other contributions, *I_tot_*=*I_pyro_* will be assumed. The *I_pyro_*(*T*) data are integrated from *T_0_* down to *T_end_*, generating an electric polarization *P_pyro_* as a function of *T*. The Pyro method is applicable only if the *P* at *T_end_* is nonzero, otherwise the electric poling down to *T_end_* is completely ineffective even if *P* is nonzero at any *T* other than *T_end_*.

SFig.1. (color online) *Schematic illustrations of the two methods used for probing the electric polarization P. (a) The Pyro method in which the released current I_tot_ of the capacitor as a function of T during the warming sequence is probed after the capacitor is cooled down to T_end_ under electric poling by a dc field E_pole_. (b) The Pole method in which the current I_tot_ flowing across the capacitor under a relatively low dc field E_pole_ is measured during the cooling sequence, here the I_tot_ includes the leakage current I_leaky_ and pyroelectric current (here it is actually the polarization current) I_pyro_, while the I_leaky_ should be as small as possible for a reliable evaluation of the I_pyro_.*

An often questioned issue for this method is that the current signals *I_tot_* possibly include contributions other than *I_pyro_*, such as electric poling induced trapped charges or/and other thermally stimulated current. A well-recognized way to exclude these contributions is to perform the measurement at several runs with different warming rates. In case of no shift between these measured *I_tot_*(*T*) curves along the *T*-axis, *I_tot_*=*I_pyro_* is recognized. This method was used in the earliest experiment on polarization of RMn_2_O_5_ [1]. Surely, due to the uncertainties associated with the apparatus, a shift of the curve as small as ~1.0K along the *T*-axis is treated as the error.

The second method is the high precision *P-E* loop (P-E) method, which is applicable for ferroelectrics with relatively large *P*. Its application to DyMn_2_O_5_ was reported, and well-defined *P-E* loops were obtained above *T_Dy_*~8K [2]. However, no identifiable loop was observed below the *T_Dy_*, by which one infers that the low-*T* phase is non-ferroelectric (the X-phase). For DyMn_2_O_5_, it was claimed that the direction of *P* is neither uniquely nor controllably fixed if the Pyro method is used [2], and this failure was thought to be related to the ineffective poling process if the *T_end_*<*T_Dy_*=8K. Surely, this P-E method may be questioned if *P* is relatively small in terms of the *P*(*T*) dependence, and the ferroelectric transition point may not be precisely determined from the *P*(*T*) data.

Based on the above discussion, Higashiyama *et al* [2] developed the third method and we may name it as the Pole method, as schematically drawn in SFig.1(b). Different from the separated poling step and probing step in the Pyro method, here the poling and probing are carried out simultaneously. The sample under an electric poling field *E_pole_* is gradually cooled down to the *T_end_* from a high *T*, during which the total current *I_tot_* across the sample is probed. This field is supposed to be small sufficiently so that the field induced leaky current *I_leaky_* is much smaller than the pyroelectric current *I_pyro_* (here it is actually the polarization current) over the whole *T*-range covered experimentally. A proper fitting procedure may allow an exclusion of the *I_leaky_*, leaving the *I_pyro_*(*T*) and thus the *P*(*T*) to be evaluated, respectively. Clearly, an immediate question regarding this Pole method is the validity of condition *I_leaky_*<<*I_pyro_*, which may not be always true even for DyMn_2_O_5_, since a small *E_pole_* implies an incomplete poling of the sample and thus the evaluated *P* may not be the saturated one. Furthermore, a separation of the *I_pyro_* from the *I_tot_* is anyway a matter if the relations *I_leaky_*(*T*) and *I_pyro_*(*T*) are unknown, while the relation *I_leaky_*(*T*) is not easily accessible. In fact, Higashiyama *et al* discussed these issues and the consequences in their work [2]. The major matter here for DyMn_2_O_5_ is that this method may not identify correctly the ferrielectric state if DyMn_2_O_5_ would be a ferrielectric.

*B. Modifying the pyroelectric current scheme*

In SFig.2(a)-(c) are plotted the measured *P*(*T*) curves for DyMn_2_O_5_ single crystals by the above mentioned three methods (Pyro, P-E, and Pole), with the corresponding polarizations denoted as *P_pyro_*, *P_P-E_*, and *P_pole_*, respectively. The boundaries between different ferroelectric phases, as given by Higashiyama *et al* [2], are marked and labeled in SFig.2(a). The measured data on polycrystalline samples by the PUND method are inserted in SFig.2(d) for comparison too.

SFig.3. (color online) *Evaluated electric polarizations as a function of T, measured by various methods, respectively. (a) The P_pyro_-T curve by the Pyro method, taken from Ref.[1], (b) the P_P-E_-T curve by the P-E hysteresis method, taken from Ref.[2], (c) the P_pole_-T curve by the Pole method, taken from Ref.[2], and (d) the P_PUND_-T curve by the PUND method in this work. Here T_N1_, T_N2_, T_N3_, and T_Dy_ are the magnetic transition points, and T_FE1_, T_FE2_, and T_FE3_, are the ferroelectric transition points, see the text. Symbol PE refers to the paraelectrics phase. Symbols FE1, FE2, and FE3, refer to the three ferroelectric phases, respectively. The X-phase, as defined in Ref.[2], refers to the claimed low-T non-ferroelectric phase.*

While no discussion on details of these measured *P*(*T*) data is given here, we only look at the correspondences between the magnetic transition points and ferroelectric transition points. The *P*(*T*) curves obtained by the Pyro method (SFig.2(a)) and the PUND method (SFig.2(d)) show anomalies roughly at *T_N1_*, *T_N2_*, *T_N3_*, and *T_Dy_*, respectively, the curves obtained by the P-E method (SFig.2(b)) and the Pole method (SFig.2(c)) show no anomalies at *T_N2_* and *T_N3_*. In particular, both the Pyro method and PUND method revealed that the X-phase is ferroelectric with considerable polarization (or possibly ferrielectric with a nonzero net polarization) at *T*<*T_Dy_*. In fact, the measured *P_P-E_*(*T*) and *P_pyro_*(*T*) curves show remarkable differences. An anomaly at *T*~13K (*T_FE3_* here) was observed in the measurements by all the P-E, Pole, and PUND methods, but not by the Pyro method, noting that only the Pyro method has zero electric bias during the measurement.

The above comparison stimulates us to revisit the Pyro method. We then modify this method to avoid the problem of ineffective electric poling. Instead of cooling the sample down to the lowest *T*, e.g. *T_end_*~2K<<*T_Dy_*, we take the *T_end_* as a variable upon request. Given that the suggested X-phase is non-ferroelectric at *T*<*T_Dy_*, we change the *T_end_* in a broad *T*-range and perform identical measurements. We name this scheme as the mPyro method which offers several advantages. Besides the aforementioned one, one may also avoid possible influence of the magnetic transitions below the *T_end_* on the multiferroic behaviors above the *T_end_*, which is believed to be significant for a number of type-II multiferroics such as MnWO_4_ [4] and RMnO_3_ (R=Gd, Dy, Ho) [5]. The possibly existing drawbacks in the P-E and Pole methods, such as leaky current contribution and uncertainties, may be excluded too.

It will be shown that the data obtained by this mPyro method are qualitatively similar to those by the Pyro and PUND methods but different from the other two. By the step-by-step varying *T_end_* up to *T_N1_* from the lowest *T* reachable, one is able to evaluate the electric polarizations in various magnetic phases, constituting the basis for sufficient discussion on the ferroelectricity in DyMn_2_O_5_.

**II. Structural characterization**

The sample crystallinity was checked using X-ray diffraction (XRD) with Cu Kα radiation at room temperature and the obtained *θ*-2*θ* spectrum is presented in SFig.3. The reflections can be well indexed by the lattice symmetry *Pbam*, as also confirmed with the refined data using the Rietveld analysis. The evaluated lattice constants are *a*=0.7298(4) nm, *b*=0.8510(5) nm, and *c*=0.5681(8) nm with factor *R_WP_*=6.41%. These data are consistent with earlier reported values [6].

SFig.4. (color online) *Measured θ-2θ XRD spectrum for the DyMn_2_O_5_ sample. The calculated one by the Rietveld analysis and the Bragg positions of the reflections are inserted for reference. The evaluated lattice constants are given in the figure too.*

**III. Demonstration of the mPyro method**

In SFig.4(a)~(c) are plotted the measured released currents using the mPyro method with *T_end_*=2K, *E_pole_*=10kV/cm, and three different warming rates, 2, 4, and 6K/min, respectively. It comes immediately to our attention that the three current-temperature curves, if normalized by the warming rate, almost perfectly overlap with each other, showing no difference between them within the measuring uncertainties less than ~0.3pA and less than 0.5K peak-to-peak shift along the *T*-axis. These peaks are sharp and well fixed while thermally stimulated currents other than the pyroelectric current are usually broad. These features indicate that the measured data do come from the pyroelectric current *I_pyro_* without identifiable contribution from other sources. In addition, the measured *I_pyro_*-*T* curve can be switched upon a reverse poling field, as shown in SFig.4(d), indicating its origin from the pyroelectricity.

SFig.4. (color online) *Measured pyroelectric current I_pyro_ as a function of T at a warming rate of 2K/min (a), a rate of 4K/min (b), and a rate of 6K/min (c), respectively. The I_pyro_-T curves measured under two opposite poling fields* ±*10kV/cm at a 2K/min warming rate as well as the evaluated polarizations P_pyro_(T) are plotted in (d) and (e) respectively.*

Moreover, the measured *I_pyro_*-*T* curves show clear anomalies at all the magnetic transition points (*T_N1_*, *T_N2_*, *T_N3_*, and *T_Dy_*), indicating the one-to-one correspondence between the magnetism and ferroelectricity. The as-evaluated *P-T* curves from the *I_pyro_*-*T* curves under *E_pole_*=±10kV/cm in SFig.4(d) are plotted in SFig.4(e). The *P-T* curve at *E_pole_*=10kV/cm is similar in shape to that reported in Ref.[1].

***References:***

1. Hur, N., Park, S., Sharma, P. A., Guha, S. & Cheong, S. W. Colossal magnetodielectric effects in DyMn_2_O_5_. *Phys. Rev. Lett.* **93**, 107207 (2004).
2. Higashiyama, D., Miyasaka, S., Kida, N., Arima, T. & Tokura, Y. Control of the ferroelectric properties of DyMn_2_O_5_ by magnetic fields. *Phys. Rev. B* **70**, 174405 (2004).
3. Lin, L. *et al*. Spin frustration destruction and ferroelectricity modulation in Ca_3_CoMnO_6_: Effects of Mn deficiency. *J. Appl. Phys.* **111**, 07D901 (2012).
4. Prokhnenko, O. *et al*. Enhanced Ferroelectric Polarization by Induced Dy Spin Order in Multiferroic DyMnO_3_. *Phys. Rev. Lett.* **98**, 057206 (2007).
5. Fukunaga, M. & Noda, Y. Measurement of complicated temperature-dependent polarization of multiferroic RMn_2_O_5_. *J. Phys.: Conference Series* **320**, 012090 (2011).
6. Ewing, R. A. *et al.* X-ray resonant diffraction study of multiferroic DyMn_2_O_5_. *Phys. Rev. B* **77**, 104415 (2008).
